# Prognostic stratification of molecularly and clinically distinct subgroup in children with acute monocytic leukemia

**DOI:** 10.1002/cam4.3023

**Published:** 2020-03-26

**Authors:** Li‐Peng Liu, Ao‐Li Zhang, Min Ruan, Li‐Xian Chang, Fang Liu, Xia Chen, Ben‐Quan Qi, Li Zhang, Yao Zou, Yu‐Mei Chen, Xiao‐Juan Chen, Wen‐Yu Yang, Ye Guo, Xiao‐Fan Zhu

**Affiliations:** ^1^ Division of Pediatric Blood Diseases Center Institute of Hematology and Blood Diseases Hospital Chinese Academy of Medical Sciences & Peking Union Medical College Tianjin China

**Keywords:** acute monocytic leukemia, children, clinical characteristics, gene mutation, prognostic factors

## Abstract

**Background:**

The prognosis of children with acute monocytic leukemia (AML‐M5) remains unsatisfactory and the risk profile is still controversial. We aim to investigate the prognostic value of clinical and cytogenetic features and propose a new risk stratification in AML‐M5 children.

**Methods:**

We included 132 children with AML‐M5. Overall survival (OS) and progression‐free survival (PFS) were documented. Cox regression was performed to evaluate the potential risk factors of prognosis.

**Results:**

The 5‐year‐OS was 46.0% (95% confidence intervals, 41.6%‐50.4%) in all patients. There was significantly lower OS in the age ≤ 3 years old (*P* = .009) and hyperleukocytosis (*P* < .001). The FMS‐like tyrosine kinase 3 (*FLT3*)‐internal tandem duplication (*ITD*) and *MLL‐rearrangement* carriers were associated with fewer survivors in all patients (37.1% and 36.7%) and chemotherapy‐only group (19.0% and 35.0%). Notably, the number of survivor with *MLL‐rearrangement* did not increase in hematopoietic stem cell transplant (HSCT) group. According to the Cox regression analysis, HSCT was a significantly favorable factor (*P* = .001), while hyperleukocytosis, age ≤ 3 years old, and BM blast ≥ 70% adversely affected the OS in all patients (all *P* < .05). Additionally, *FLT3‐ITD* was a risk factor for OS in the chemotherapy‐only group (*P* = .023), while hyperleukocytosis and age ≤ 3 years independently contributed to poor PFS (both *P* < .05). In comparison to the standard‐risk group, significant poorer outcome was found in the high‐risk group (both *P* < .005).

**Conclusions:**

We propose that AML‐M5 children with any of *MLL‐rearrangement*, *FLT3‐ITD*, hyperleukocytosis, BM blast ≥ 70%, or age ≤ 3 years old are classified into the high‐risk group, and HSCT is beneficial especially in patients with *FLT3‐ITD* mutation, hyperleukocytosis, and age ≤ 3 years old. Importantly, the choice of HSCT should be made more carefully in children with *MLL‐rearrangement* for its suboptimal performance.

## Graphical abstract

Outcomes in high‐risk acute monocytic leukemia‐M5 children with only conventional chemotherapy remain unsatisfactory, and hematopoietic stem cell transplant (HSCT) has proven to be beneficial especially in FMS‐like tyrosine kinase 3‐internal tandem duplication carriers, age ≤ 3 years old and hyperleukocytic patients. The choice of HSCT should be made more carefully in a defined subgroup with *MLL‐R* for its suboptimal performance.

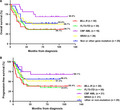

## 1 INTRODUCTION

Acute monocytic leukemia (AML)‐M5 is one of the common types of AMLs defined as M5 in the French‐American‐British classification. Nevertheless, the prognosis of patients with AML‐M5 remains unsatisfactory, for the 3‐year disease‐free survival rate was 26% and overall survival (OS) rate was only about 31%.^1^

The intensity of therapy should be tailored to the risk profile of pediatric AML. Chemotherapy alone is favored in low‐risk AML, whereas allogeneic hematopoietic stem cell transplantation (HSCT) is favored in high‐risk AML. However, the treatment approach is more controversial in children with AML‐M5 because of its poor prognosis.^2^, ^3^ Apart from response to treatment, the most important factor in predicting prognosis of AML patients is cytogenetic aberrations.^4^ With the increased development of the techniques in molecular biology, it is feasible to generate the examination of outcome and classify different entities. Especially, after the application of next generation sequencing (NGS), this emerging technology has narrowed the gap of knowledge in the molecular biology of pediatric AML by discrimination of targeted gene mutations for separate subtype, which may led to the improvement in terms of prognosis prediction and the use of specific and therapeutic intervention.^5^ Moreover, identification of major cytogenetic abnormalities (including genome, transcriptome, and epigenome) defined by new technologies allowed more precise risk stratification for intermediate‐risk‐AML.^6^

For example, the well‐known genetic alterations that could evaluate the prognosis are inv(16) (p13q22) (*CBFβ‐MYH11*), t(8;21)(q22;q22) (*AML1‐ETO*) and mixed lineage leukemia rearrangements (*MLL‐R*). Other mutations, such as FMS‐like tyrosine kinase 3 (*FLT3*)‐internal tandem duplication (*ITD*), were also correlated with worse prognosis of AMLs.^7^, ^8^ Pediatric AML patients often presented with *FLT3‐ITD* and *MLL‐R*, which lead to a poor prognosis.^9^ Moreover, other clinical features, such as hyperleukocytosis, chromosomal karyotypes, blast percentage in bone marrow (BM), and age at first diagnosis, may also closely associate with the short‐ or long‐term prognosis in AML children.^10^, ^11^

To our knowledge, no recently published research has assessed the prognostic factors for AML‐M5 children, and the influence of the certain clinical features in a cohort with pediatric individuals. Hence, we purport to investigate the impact of clinical characteristics and mutational signatures as prognostic markers in pediatric AML‐M5 and proposed a new risk stratification for these patients.

## 2 MATERIALS AND METHODS

### 2.1 Study population

The source population for the study included 132 children with AML‐M5 at the Institute of Hematology and Blood Diseases Hospital, Chinese Academy of Medical Sciences & Peking Union Medical College between January 2005 and January 2015. Patients with Down syndrome were excluded. The data collected included information regarding age, sex, peripheral blood (PB) white blood cell counts (WBC), blast percentages in BM, chromosome karyotypes, and gene mutation signatures.

The study design and methods complied with the Declaration of Helsinki and was approved by the Ethics Committee and Institutional Review Board of Institute of Hematology and Blood Diseases Hospital, Chinese Academy of Medical Sciences & Peking Union Medical College. Informed consent was obtained from all subjects.

### 2.2 Data collection and definitions

All patients were assessed every 6 months after completion of all treatment and those who did not visit our division were followed up by telephone. Patients who lost contact from completion of treatment were defined as lost to follow‐up. Hyperleukocytosis was defined as WBC counts >100 × 10^9^/L. Complete remission (CR) was regarded as the combination of morphologic remission (<5% BM blasts) and absence of a unique phenotype by flow cytometry after induction. Relapse was defined as the return of leukemia cells in the BM after CR acquisition or gaining evidence of leukemia cell infiltration in other tissues or organs. Progression‐free survival (PFS) and OS during follow‐up were the primary endpoints of this study. Progression‐free survival was defined as the interval between initial treatment and the first documentation of disease progression or death. OS was defined as the time from diagnosis to death or was censored at the last follow‐up. AML‐M5 patients is characterized by an overwhelming number of immature monocytic cells (>80% of monocytic cells) in the BM and PB.

### 2.3 Chemotherapy regimen

All patients in this study received Chinese Academy of Medical Science (CAMS)‐2009 regimen. In brief, the induction therapy includes a combination of etoposide 150 mg/m^2^ with a 2 hour infusion on days 1‐5, idarubicin, 8 mg/m^2^ iv on days 6‐8 and a 12 hour infusion of cytarabine (Ara‐C), 200 mg/m^2^ on days 6‐12. A second course of induction treatment was given if CR had still not been achieved. Patients entering CR were scheduled to receive five courses of consolidation therapy. Course 1 and 4 were: idarubicin, 10 mg/m^2^ days 1 and Ara‐C 3 g/m^2^ 12 hourly, days 1‐3. Course 2 and 5 was mitoxantrone 5 mg/m^2^ days 4‐6, Ara‐C 200 mg/m^2^ with a 24 hour infusion days 4‐8, and etoposide 150 mg/m^2^ with a 2 hour infusion days 1‐3. Course 3 was Ara‐C 2 g/m^2^ with a 3 hour infusion days 1‐5 and etoposide 100 mg/m^2^ with a 2 hour infusion days 1‐5. FMS‐like tyrosine kinase 3‐internal tandem duplication or ≥15% blasts after first induction or ≥5% blasts on morphology or ≥0.1% by flow cytometry after second induction, and relapsed patients are the potential indications for HSCT. However, selection for and timing of HSCT also depends on the condition of patients’ guardians and donors.

### 2.4 Genetic mutation analysis

The genetic mutation status of all patients was analyzed at the time of admission. Genomic DNA was extracted using the EZNA blood DNA Midi Kit (Omega Bio‐Tek). Whenever possible, BM samples were used for analyses. DNA samples were sequenced using the MiSeq platform (Illumina), which is a custom, targeted, amplicon‐based sequencing approach by NGS. Libraries were prepared with a custom amplicon panel targeting *AML1/ETO* (*RUNX1*/*RUNX1T1*), *CBFβ‐MYH11*, *DNMT3A*, *ETNK1*, *ETV6*, *EZH2*, *FLT3*, *GATA2*, *IDH1*, *IDH2*, *IL7R*, *JAK2*, *ASXL1*, *ASXL2*, *BCOR*, *BCORL1*, *BIRC3*, *BRAF*, *CALR*, *CBL*, *CDKN2A*, *KIT*, *KMT2A* (*MLL*), *KRAS*, *MPL*, *MYD88*, *NOTCH1*, *NRAS*, *PAX5*, *PDGFRA*, *PDGFRB*, *PTEN*, *PTPN11*, *SETBP1*, *SETD2*, *STAG2*, *TET2*, *TP53,* and *WT1*, with a median depth of 2000×.

### 2.5 Statistical analysis

Kolmogorov‐Smirnov normality test was performed to examine whether the data showed normal distribution or not. The result indicated that all quantitative data do not comply with the normal distribution. Datasets were described with median and/or range. Differences in continuous variables were analyzed using the Mann‐Whitney *U* test, and categorical data were analyzed using Pearson chi‐squared analysis. Survival rates were estimated using the Kaplan‐Meier method and compared using the log‐rank test. A cox regression model was to evaluate risk factors for PFS and OS of all patients. For univariate analysis, the results were presented as odds ratio (OR), 95% confidence intervals (CI), and *P* value. All variables with a *P* < .10 in univariate analysis were included in the multivariate analysis in logistic regression and Cox regression model. Two‐tailed *P* < .05 was considered statistically significant. All statistical analysis was performed using SPSS 22.0 (IBM Corporation) and GraphPad Prism 8.02 software (GraphPad Software Inc).

## 3 RESULTS

### 3.1 Baseline characteristics

During the study period, we identified 132 children with AML‐M5 at the Institute of Hematology and Blood Diseases Hospital, Chinese Academy of Medical Sciences & Peking Union Medical College, and the median follow‐up was 34 months (1032 days). Clinical and molecular characteristics of all patients were summarized in Table 1, the median patient age was 6 years (range 1‐17) and 84 patients (63.6%) were male. Age ≤ 3 years old (n = 37) and patients with hyperleukocytosis (n = 55) comprised 28.0% and 41.7%, respectively, of all patients. About 75 (56.8%) patients had BM blast percentage ≥ 70% and 50 (37.9%) patients had abnormal karyotypes.

**TABLE 1:** Clinical characteristics of patients with acute myeloid leukemia‐M5

	HSCT (N = 44)	Non‐HSCT (N = 88)	*P* value
Age (y), median (range)	7 (1‐17)	6 (1‐14)	0.677
Male, no. (%)	27 (61.4)	57 (64.8)	0.701
Age ≤ 3 y old, no. (%)	12 (27.3)	25 (28.4)	0.891
WBC> 100 × 10^9^/L, no. (%)	22 (50.0)	33 (37.5)	0.170
Complex karyotype, no. (%)	25 (56.8)	49 (55.7)	0.901
Cytogenetic, mutation group, no. (%)			
*MLL‐R*	10 (22.7)	20 (22.7)	0.999
*FLT3‐ITD*	14 (31.8)	21 (23.9)	0.329
*NRAS*	5 (11.4)	21 (23.9)	0.089
CBF‐AML	6 (13.6)	10 (11.4)	0.706
No gene mutation or other mutation	9 (20.5)	16 (18.2)	0.753

Abbreviations: CBF‐AML, Core binding factor acute myeloid leukemia (*AML1‐ETO* or *CBFβ/MYH11*); HSCT, hematopoietic stem cell transplantation.

In particular, *FLT3‐ITD* was the most frequent molecular alteration in all patients (n = 35), followed by *MLL‐R* (n = 30), *NRAS* (n = 26) and *AML1/ETO* or *CBFβ/MYH11* (n = 16), while 25 patients had no mutation or other mutation of the genes. Among the population, 88 patients underwent chemotherapy‐only and 44 also underwent allo‐HSCT. Table 1 illustrated that there exist insignificant difference between the HSCT and non‐HSCT groups in terms of baseline characteristics.

### 3.2 Prognostic analysis of the patients

**FIGURE 1:**
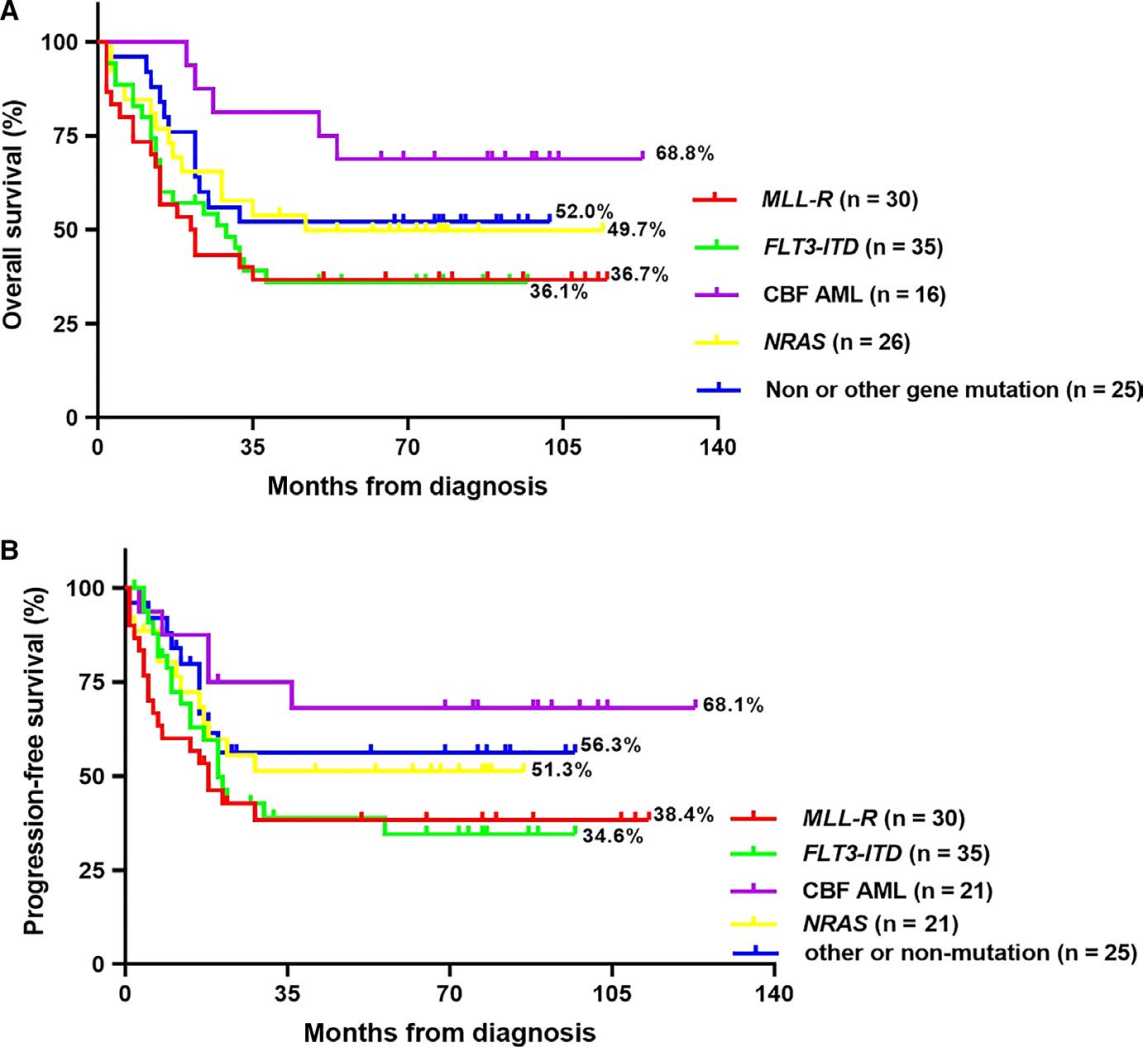
A and B, Prognosis analysis of all children with acute monocytic leukemia (AML)‐M5 according to the presence of molecular alterations: Prognostic analysis of 132 AML‐M5 children was performed using four of the most common fusion genes. Children with CBF‐AML (*AML1/ETO* or *CBFβ/MYH11*) had the best prognosis regarding overall survival (OS) and progression‐free survival (PFS). Notably, children with *NRAS* had intermediate outcomes concerning OS and PFS, whereas patients with *MLL‐R*, *FLT3‐ITD* had the poorer OS and PFS

With a median follow‐up time among survivors of 34 months (range: 2‐123 months), the estimated 5‐year OS rate was 46.0% (95% CI, 41.6%‐50.4%) in all AML‐M5 children, and the HSCT group's OS was significantly higher than the non‐HSCT group (58.3 ± 7.5% vs 39.8 ± 5.2%, *P* = .008). Moreover, there was a significantly lower OS in the infants (age ≤ 3 years) in comparison to children >3 years (28.5 ± 7.5% vs 52.5% ± 5.1%, *P* = .009) (Figure S1A), and poorer OS in the patients with hyperleukocytosis when compared with non‐hyperleukocytosis (23.6 ± 5.7% vs 60.5 ± 5.8%, *P* < .001) (Figure S2A). Moreover, according to the Kaplan‐Meier analysis, there was also significantly lower PFS in patients ≤3 years old and with hyperleukocytosis (both *P* < .05) (Figures S1B and S2B). As for patients with separate genetic alterations are concerned, Figure 1A indicated that the *MLL‐R* and *FLT3‐ITD* mutation's presence was associated with poorer prognosis while CBF‐AML (*AML1/ETO* or *CBFβ/MYH11*) was with better prognosis in the total cohort.

**FIGURE 2:**
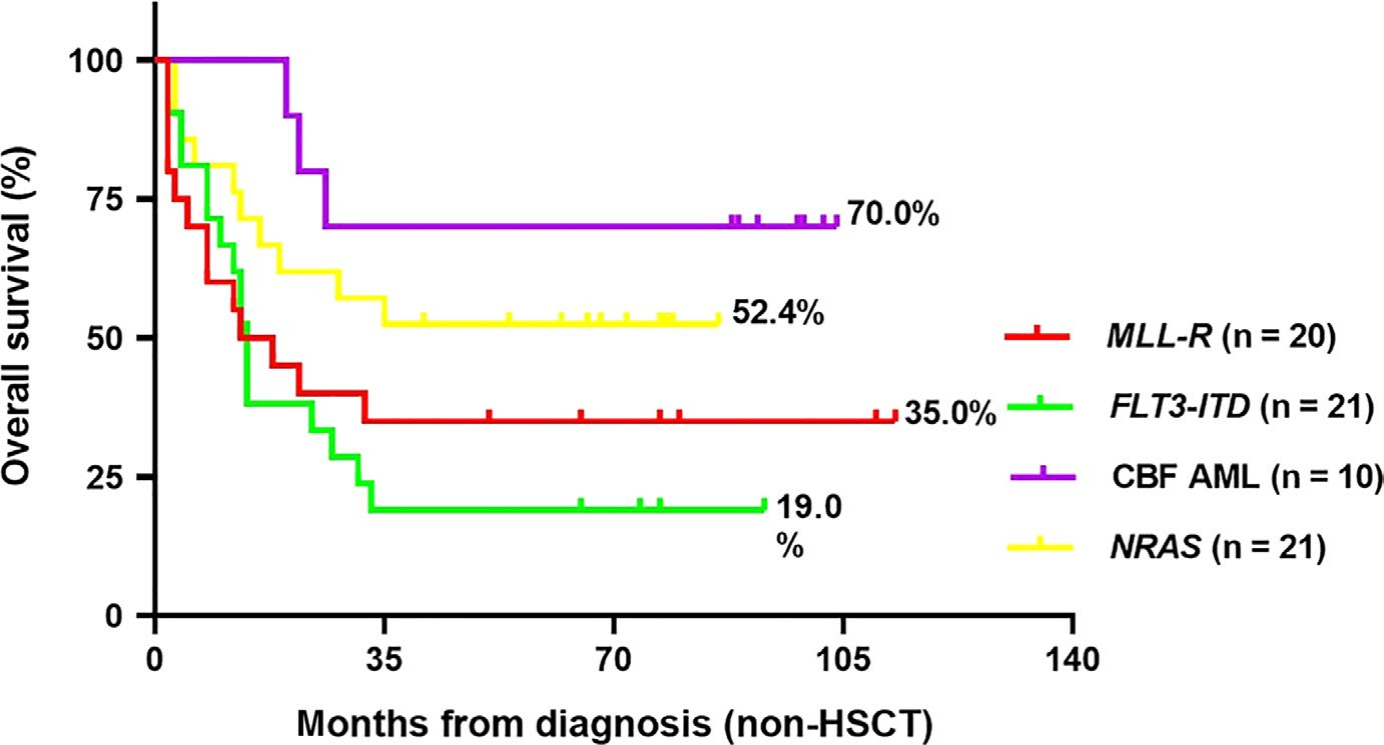
Overall survival (OS) of chemotherapy‐only children with acute monocytic leukemia (AML)‐M5 according to the presence of molecular alterations: prognostic analysis of 88 non‐hematopoietic stem cell transplant (HSCT) AML‐M5 children was performed using four of the most common fusion genes. Children with CBF‐AML (*AML1/ETO* or *CBFβ/MYH11*) had the best prognosis regarding OS, while children with *NRAS* and *MLL‐R* had intermediate prognosis, whereas patients with *FLT3‐ITD* had the poorer outcome

In consistent with the result of OS, Figure 1B illustrated that PFS of *MLL‐R* and *FLT3‐ITD* carriers was generally poorer than other patients. Then, we confined the subject to the 88 patients who did not receive HSCT (Figure 2). Surprisingly, the OS of 21 non‐HSCT patients who carried *FLT3‐ITD* was significantly decrease further when compared with non *FLT3‐ITD* carriers in all individuals (19.0 ± 8.6% vs 46.3 ± 8.1%, *P* = .019). However, we did not find an obvious difference in OS among other gene mutations.

### 3.3 Analysis of potential prognostic factors of AML‐M5 children

In all 132 patients, results of multivariate Cox regression analysis revealed that hyperleukocytosis was a significant poor prognostic factor (OR [95% CI] 2.43 [1.39‐4.23]; *P* = .002), while HSCT was a significant beneficial factor (OR [95% CI] 0.45 [0.26‐0.80]; *P* = .006) for OS (Table 2). According to univariate analysis, hyperleukocytosis (*P* < .001), age ≤ 3 years (*P* = .008), BM blast percentage ≥ 70% (*P* = .008), and *FLT3‐ITD* mutation (*P* = .023) were significantly associated with poor outcome in non‐HSCT group. The result of the multivariate analysis was also shown in Table 2, and only hyperleukocytosis significantly related to the OS (*P* = .001).

**TABLE 2:** Univariate and multivariate analysis for risk factors of overall survival (OS) and progression‐free survival (PFS) in all patients

	Univariate analysis		Multivariate analysis	
	OR (95% CI)	*P* value	OR (95% CI)	*P* value
OS of all patients (n = 132)				
Age ≤ 3 y	1.88 (1.16‐3.05)	0.011	1.51 (0.92‐2.50)	0.105
BM blast ≥ 70%	1.78 (1.07‐2.97)	0.026	1.38 (0.82‐2.34)	0.229
Karyotype	1.62 (1.01‐2.61)	0.045	1.35 (0.83‐2.21)	0.229
Hyperleukocytosis	2.78 (1.72‐4.48)	<0.001	2.82 (1.68‐4.74)	<0.001
HSCT	0.49 (0.29‐0.84)	0.010	0.39 (0.22‐0.67)	0.001
OS of non‐HSCT patients (n = 88)				
Age ≤3 y	2.12 (1.22‐3.71)	0.008	1.51 (0.82‐2.77)	0.182
BM blast ≥ 70%	2.46 (1.26‐4.79)	0.008	1.81 (0.89‐3.67)	0.101
Karyotype	1.58 (0.91‐2.71)	0.102	—	—
Hyperleukocytosis	3.32 (1.91‐5.78)	<0.001	2.66 (1.47‐4.84)	0.001
*FLT3‐ITD*	1.96 (1.10‐3.50)	0.023	1.30 (0.69‐2.45)	0.421
*MLL‐R*	1.39 (0.74‐2.60)	0.305	—	—
PFS of all patients (n = 132)				
Age ≤3 y	2.10 (1.27‐3.48)	0.004	1.73 (1.04‐2.88)	0.036
BM blast ≥ 70%	1.56 (0.92‐2.62)	0.097	1.29 (0.76‐2.19)	0.342
Karyotype	1.56 (0.95‐2.55)	0.078	1.21 (0.73‐2.02)	0.457
Hyperleukocytosis	3.04 (1.83‐5.03)	<0.001	2.62 (1.54‐4.45)	<0.001

Abbreviations: Blast percentages, blast percentages in bone marrow at first diagnosis; CI, confidence interval; HSCT, hematopoietic stem cell transplantation; OR, odds ratio; OS, overall survival; PFS, progression‐free survival.

When it comes to prognostic factors for PFS, univariate analysis indicated that age ≤ 3 years (OR [95% CI] 2.10 [1.27‐3.48]; *P* = .004) and hyperleukocytosis (OR [95% CI] 3.04 [1.83‐5.03]; *P* < .001) adversely affected the PFS of all patients. Multivariate analysis showed that age ≤ 3 years and hyperleukocytosis were independent risk factors for PFS (both *P* < .05).

### 3.4 Influence of clinical characteristics and gene mutations on CR and relapse rate

After induction chemotherapy, 57/132 (43.2%) of all AML‐M5 children achieve a CR, and only 61/132 (46.2%) of them remain alive before the last follow‐up. The *FLT3‐ITD* and *MLL‐R* mutation's presence was associated with lower rate of CR (31.4% and 20.0%), higher recurrence (57.1% and 60.0%), and fewer survivor (37.1% and 36.7%) in comparison to the other patients. Similarly, to the *FLT3‐ITD* and *MLL‐R* cases, patients with hyperleukocytosis and age ≤ 3 years were also with low CR rate and poor prognosis. Conversely, *NRAS* and CBF‐AML (*AML1‐ETO* or *CBFβ/MYH11*) carriers experienced higher CR rate and better prognosis in all patients. More importantly, there was no significant increase in the number of survivors in HSCT group with *MLL‐R* mutation (*P* = .598).

In 88 patients who did not receive HSCT, there was significantly fewer survivor in subgroup of *FLT3‐ITD* mutation (19.0%) together with hyperleukocytosis (9.1%) and age ≤ 3 years (20.0%) among all non‐HSCT children. In view of this, no matter in all individuals or non‐HSCT subgroup, *FLT3‐ITD* carrier, age ≤ 3 years, and hyperleukocytosis could be regarded as independent adverse factors in AML‐M5 children. (Table 3).

**TABLE 3:** Outcome of patients with different genetic subtypes and clinical features

	Total	*FLT3*‐*ITD*	*MLL*‐*R*	*NRAS*	CBF‐AML	Hyperleukocytosis	Age ≤ 3 y old
CR of all patients	57/132 (43.2%)	11/35 (31.4%)	6/30 (20.0%)	16/26 (61.5%)	12/16 (75.0%)	13/55 (23.6%)	11/37 (29.7%)
Relapse of all patients	65/132 (49.2%)	20/35 (57.1%)	18/30 (60.0%)	12/26 (46.2%)	5/16 (31.2%)	40/55 (72.7%)	25/37 (67.6%)
Survivor of all patients	61/132 (46.2%)	13/35 (37.1%)	11/30 (36.7%)	13/26 (50.0%)	11/16 (68.8%)	13/55 (23.6%)	11/37 (29.7%)
Survivor without HSCT	35/88 (39.8%)	4/21 (19.0%)	7/20 (35.0%)	11/21 (52.4%)	7/10 (70.0%)	3/33 (9.1%)	5/25 (20.0%)

Abbreviations: CBF‐AML, Core binding factor acute myeloid leukemia (*AML1‐ETO* or *CBFβ/MYH11*); CR, achieved a complete response (CR) after the first induction therapy.

### 3.5 Proposal of the new risk classification for the AML‐M5 children

**FIGURE 3:**
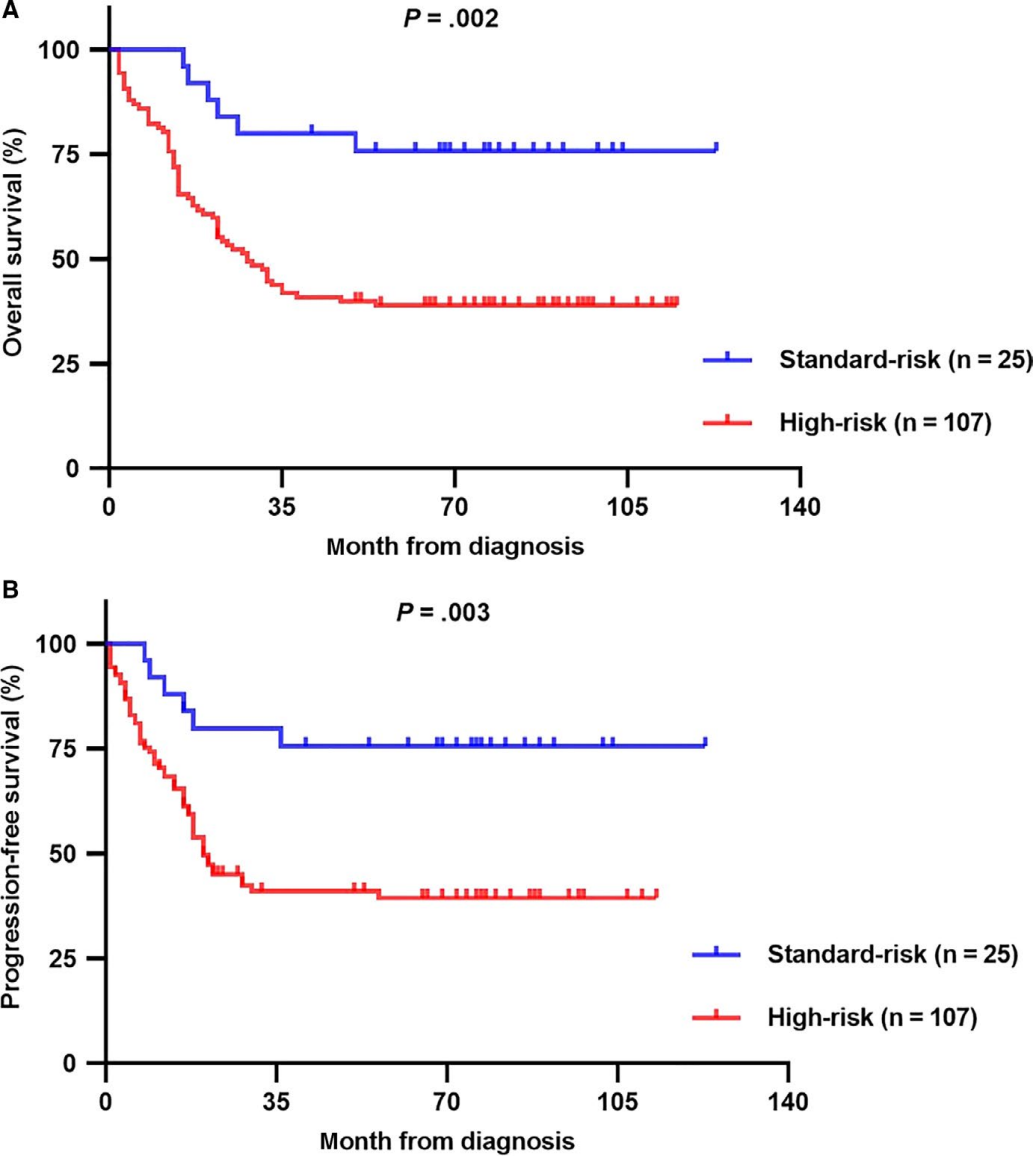
A and B, Kaplan‐Meier analysis for the proposed risk stratification of acute monocytic leukemia (AML)‐M5 children in the two risk groups. Patients with any of *MLL‐rearrangement*, *FLT3‐ITD*, hyperleukocytosis, BM blast ≥ 70%, or age ≤ 3 y old are classified into the high‐risk group (n = 107), and patients without these features were classified into the standard‐risk group (n = 25). In comparison to the standard‐risk group, significant poorer OS (*P* = .002) and PFS (*P* = .003) were found in the high‐risk group

A new risk classification for the AML‐M5 children in the two risk groups (standard‐risk and high‐risk) was presented in Figure 3. On basis of the prognostic analysis, patients with any of *MLL‐R*, *FLT3‐ITD*, hyperleukocytosis, BM blast ≥ 70%, or age ≤ 3 years old were classified into the high‐risk group (n = 107). Meanwhile, patients without these abnormalities were classified as the standard‐risk group (n = 25). Significant differences were observed in terms of OS and PFS (both *P* < .005). The CR rate was 84.0% (21/25) in the standard‐risk group, which was significantly higher than 33.6% (36/107) in the high‐risk group (*P* < .001).

## 4 DISCUSSION

The complex interaction between gene mutations and clinical features in AML have been reported by previous studies.^12^ But the effect of detailed mutation genes and certain clinical characteristics on prognosis of pediatric AML‐M5 is still lacking, moreover, comprehensive risk classification is yet to be defined. To the best of our knowledge, this study represents the first attempt to introduce clinical characteristics and molecular alterations as prognostic factors in AML‐M5 children, and propose a potential risk stratification for this population.

AML‐M5, a subtype of AML, affects mostly young children and has a poor prognosis. Although the treatment of the past 10 years has been greatly improved, the most recent studies have indicated only the modest improvement in the prognosis of AML‐M5 in childhood.^13^ Therefore, further refinement of relevant clinical and gene abnormalities in different subgroups might ultimately result in more individualized treatment regimens and potentially improve outcome.^14^

In our study, the most commonly mutated genes were *FLT3‐ITD*, *MLL‐R*, *NRAS*, *AML1/ETO*, and *CBFβ‐MYH11*, which was in accord with the majority of studies.^15^, ^16^, ^17^, ^18^ Compared to other forms of mutation, CBF‐AML (*AML1/ETO* or *CBFβ‐MYH11*) has a relatively good prognosis: about 75% of AML‐M5 individuals achieved CR following the induction therapy, compared with only 43.2% of those in all patients. Thus, CBF‐AML‐M5 children are also considered to have a favorable prognosis as previous study indicated.^19^

According to previous studies, the frequency of *NRAS* mutation was several times higher than that of *KRAS* mutation in AML, and children with M5 subsets were often associated with *RAS* mutation.^20^, ^21^ Studies of the USA Children's Oncology Group and Japanese Childhood AML Cooperative Study Group reported that *NRAS* mutation did not show poor prognosis in pediatric AMLs in Japan and US.^22^, ^23^ However, the findings in AML‐M5 children have not been disclosed so far. In this study, we found that *NRAS* mutation did not affect the outcome in pediatric AML‐M5s, as we investigate better prognosis and CR rate in patients with the mutated *NRAS*.

FMS‐like tyrosine kinase 3‐internal tandem duplication is one of the common mutations in pediatric AML and with poor prognosis.^24^ Even recently, *FLT3‐ITD* AML patients exhibited just limited progress in prognosis notwithstanding the more intensive chemotherapy according to different studies.^25^ Therefore, the current standard of care for AML children with *FLT3‐ITD* mutation is intensive chemotherapy plus a tyrosine kinase inhibitors (TKI), such as sorafenib, midostaurin, or quizartinib, followed by HSCT.^26^ Our finding is similar to previous studies, we found that *FLT3‐ITD* was the most frequent molecular alteration in AML‐M5 children, and associated with high relapse rate and poor outcome, when treated with chemotherapy alone. *FLT3‐ITD* patients who underwent allogeneic HSCT, by contrast, were associated with significantly favorable prognosis in this study. In view of this, HSCT was with a potentially beneficial effect in *FLT3‐ITD* AML‐M5 children so as to improve the outcome.

Hyperleukocytosis is associated with probability of severe complications, which may lead to tumor lysis syndrome, renal failure, or disseminated intravascular coagulopathy.^27^ Thus, hyperleukocytic patients share an increased risk of higher mortality during the treatment, especially in the initial induction of antileukemic therapy. In this study, approximately 41.7% of patients present with hyperleukocytosis at diagnosis, and these children were associated with shorter OS and PFS.

It is known that some clinical features may not play a decisive role in stratification of AML patients, however, some gene alterations are likely to work in a specific manner with a status of certain clinical feature.^28^ In this study, patients ≤3 years old and higher BM blast percentage were regarded as independent risk factors for OS and PFS. Since the close association between these clinical characteristics with hyperleukocytosis and *FLT3‐ITD* mutation, these factors were also expected to be potential prognostic markers in AML‐M5 children. Compared to other forms of acute myeloid leukemia, CBF‐AML has a relatively good prognosis: about 75% of individuals with CBF‐AML achieved a CR following treatment, compared with 43.2% of those with other forms of AML‐M5 children in this study.

Although the high incidence of *MLL‐R* in the case of children AML, the relationship between *MLL‐R* and outcome in AML is less straightforward than in ALL.^29^ Hara et al reported that *MLL‐R* has age‐specific prognostic effect, and the children ≤3 years with this fusion gene had a better prognosis than the older ones.^30^ Conversely, when analyzing a large cohort of pediatric AMLs with *MLL‐R*, 5‐year EFS and OS were poorer, when compared with all pediatric AML.^31^ In our study, although *MLL‐R* is an adverse prognostic factor for all patients, we did not find the significant age‐specific difference on prognosis in all carriers. More importantly, there was no significant increase in the number of survivors in HSCT group with *MLL‐R* mutation at any age group. The role of HSCT in the treatment of AML with *MLL‐R* is still controversial, with several studies reporting that HSCT does not improve the OS rate in *MLL‐R* adolescent AML.^32^, ^33^ In view of this, the choice of HSCT should be made more carefully in a defined subgroup of AML‐M5 children with *MLL‐R*, particularly if the donor choice and conditioning regimen not allow for a low transplant‐related mortality.

On basis of the clearly different prognosis in certain molecular and clinical characteristics, we proposed a new risk stratification for AML‐M5 children. Significant poorer OS and PFS were observed in high‐risk patients with any of *MLL‐R*, *FLT3‐ITD*, hyperleukocytosis, BM blast percentage ≥ 70%, or age ≤ 3 years. The poor outcome in high‐risk group sees the great need for refined combination chemotherapy with new agents (such as azacytidine and gemtuzumab ozogamicin et al) to intensify treatment, however, the choice of HSCT in *MLL‐R* mutation carriers required serious reconsideration. In the standard‐risk group, although the patients are vulnerable to aggressive chemotherapy and with better prognosis, children with *FLT3‐ITD* mutation and hyperleukocytosis may benefit from timely HSCT.

There is also a limitation in our study. We did not distinguish the specific subtype of *MLL‐R*. According to Meyer et al, a total of 135 different type of *MLL‐R* have been identified so far. The rearrangements of the *MLL* gene differed significantly in the separate cohorts of leukemia patients, such as infant, pediatric, or adult, moreover, the rearrangements also significantly correlated with age or gender at diagnosis.^34^ So we fail to give an identification of different fusion partners, and further papers are warranted to validate the effect of this molecular alteration.

In conclusion, we consider that this study will contribute to modify the risk stratification of childhood AML‐M5, leading to specific treatment, and improved prognosis. Despite some progress than before, outcomes in high‐risk AML‐M5 children with only conventional chemotherapy remain unsatisfactory, and HSCT may be with a beneficial effect in *FLT3‐ITD* carriers, age ≤ 3 years old, and hyperleukocytic patients. However, the choice of HSCT should be made more carefully in a defined subgroup with *MLL‐R* for its suboptimal performance.

## CONFLICT OF INTEREST

None declared.

## AUTHOR CONTRIBUTIONS

Conception and design: LLP, YG, XFZ; development of methodology: XJC, WYY, MHY, MR, XC; acquisition of data: FL LXC, BQQ, YZ; writing, review, and/or revision of the manuscript: LLP, ALZ, LZ, YMC.

## Supporting information

Fig S1A

Fig S1B

Fig S2A

Fig S2B

Supplementary Material

## Data Availability

All raw data of this study were uploaded as a supplementary material.
