# Supplementary figures and images for: Prognostic stratification of molecularly and clinically distinct subgroup in children with acute monocytic leukemia
Source: Cancer Med. 2020 Mar 26;9(11):3647–55. doi: 10.1002/cam4.3023 (PMC7286455; doi:10.1002/cam4.3023)

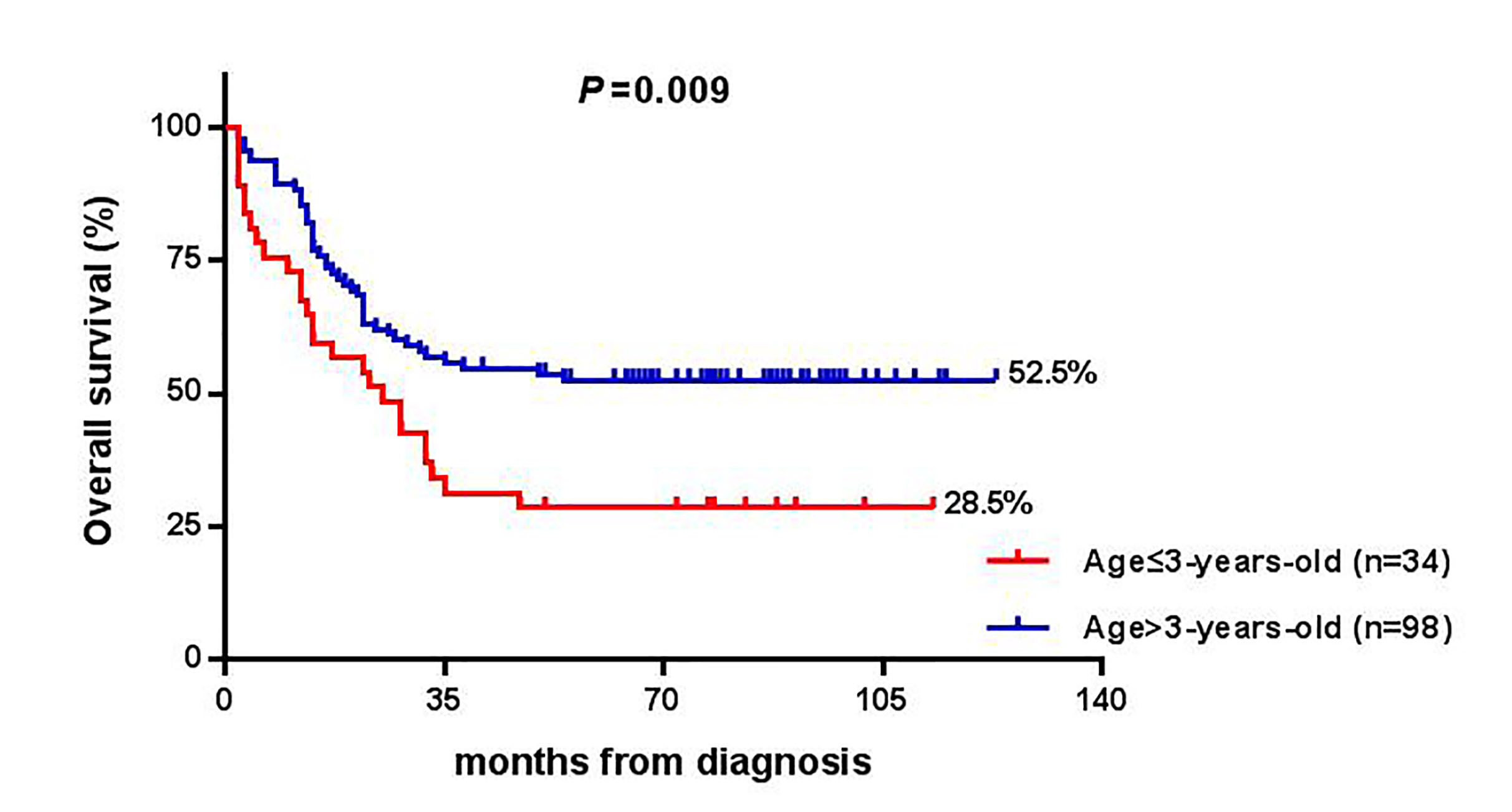

Supplement: Supplementary file 1 — Fig S1A [file CAM4-9-3647-s001.jpg]

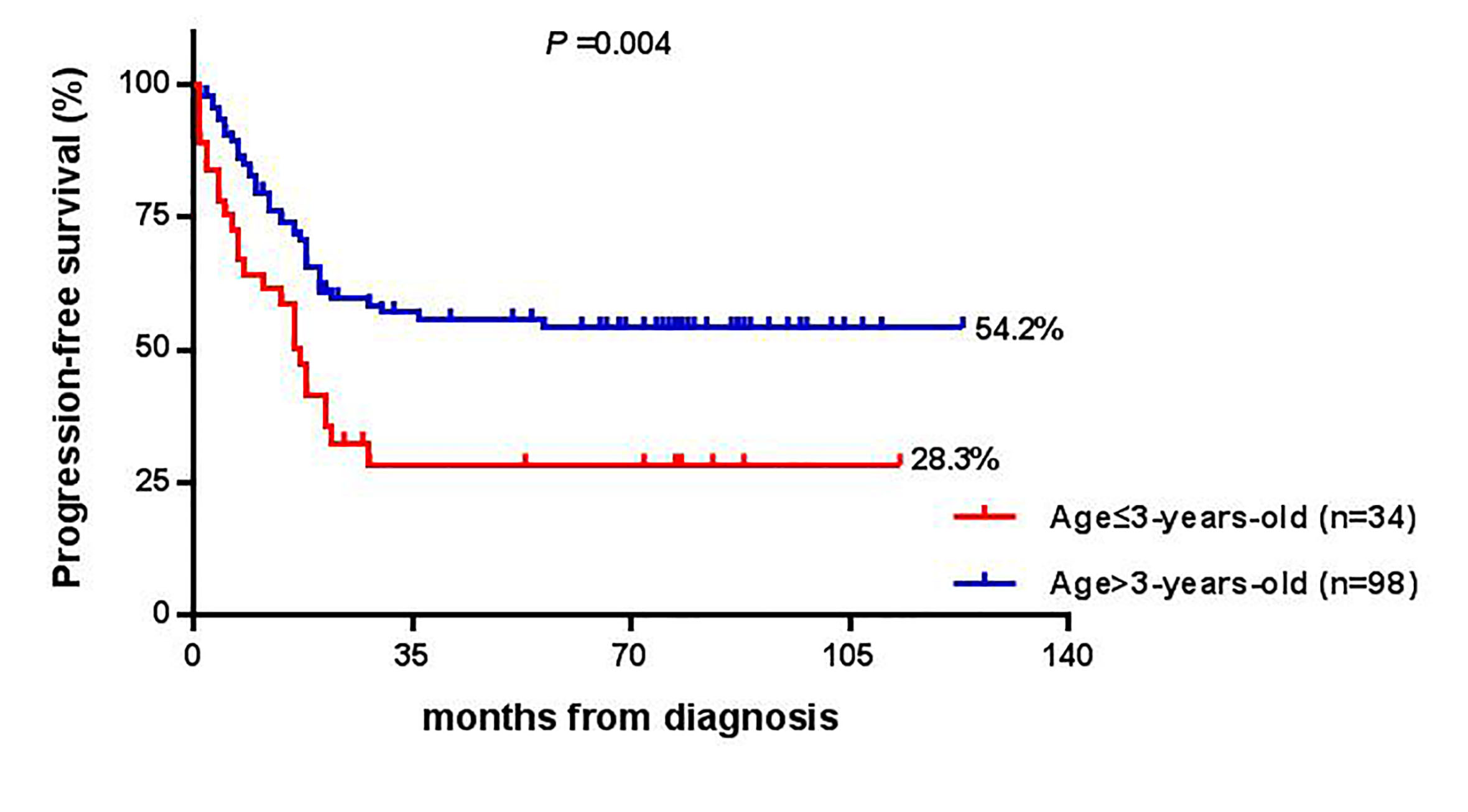

Supplement: Supplementary file 2 — Fig S1B [file CAM4-9-3647-s002.jpg]

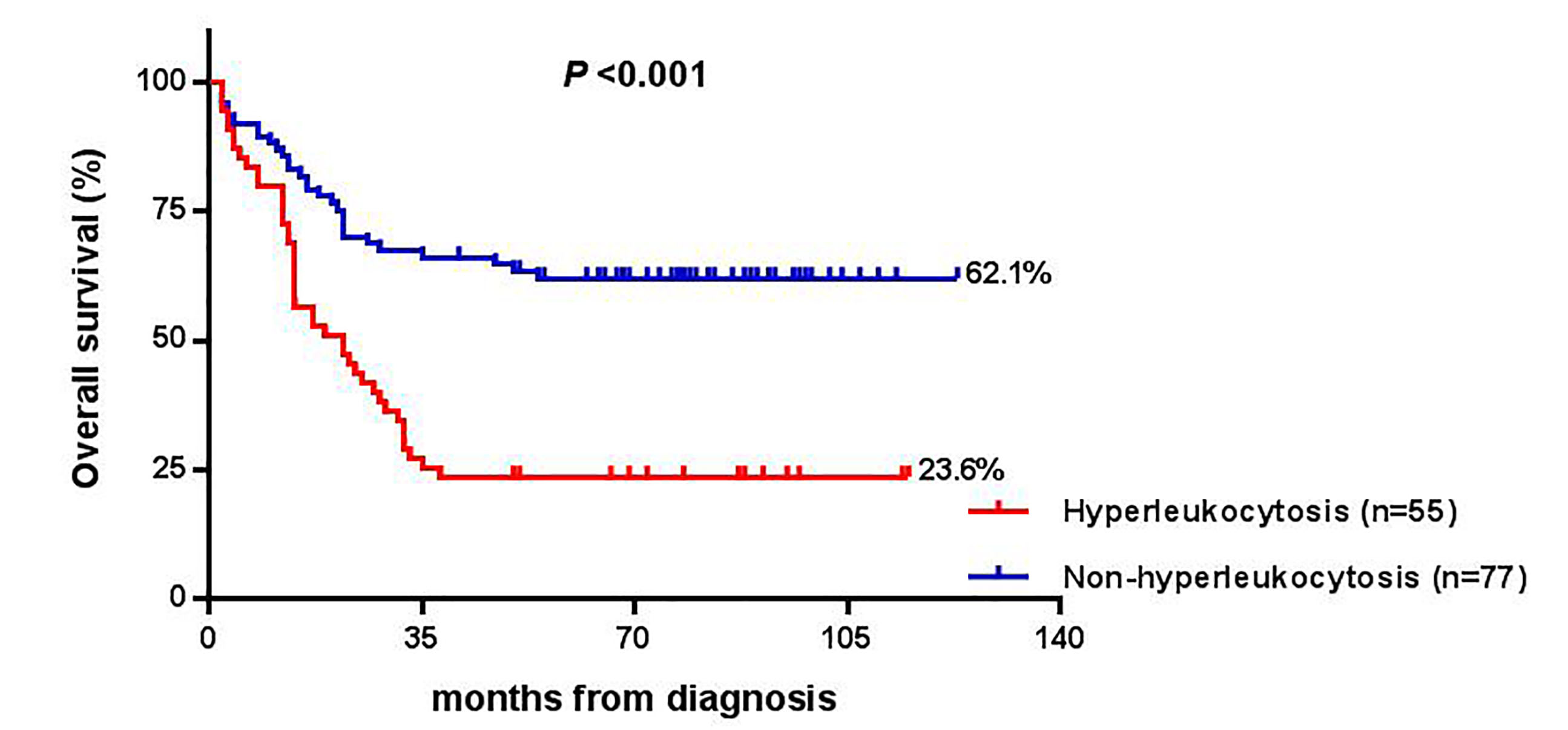

Supplement: Supplementary file 3 — Fig S2A [file CAM4-9-3647-s003.jpg]

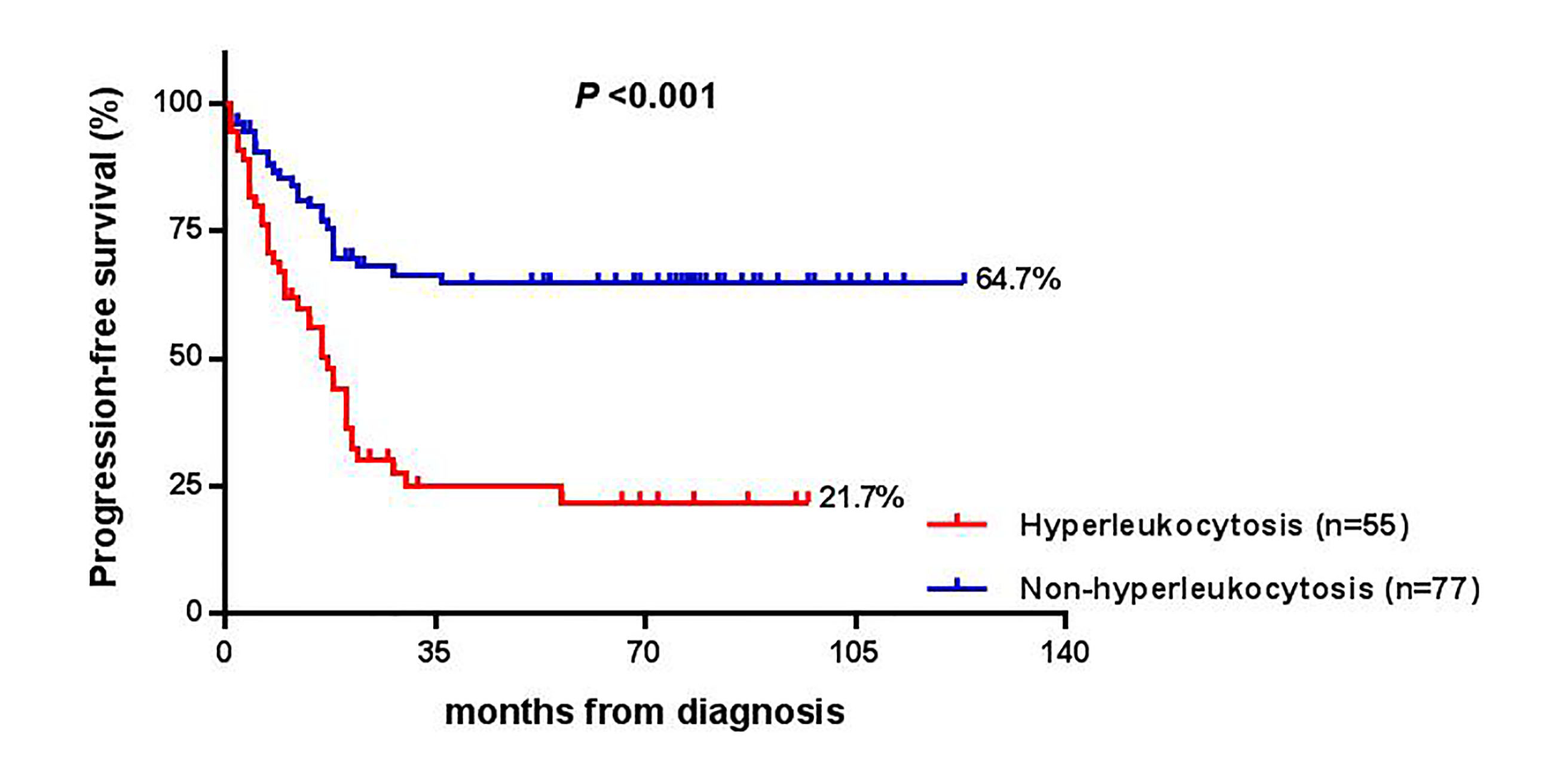

Supplement: Supplementary file 4 — Fig S2B [file CAM4-9-3647-s004.jpg]
