# Supplementary material for: Prognostic stratification of molecularly and clinically distinct subgroup in children with acute monocytic leukemia
Source: Cancer Med. 2020 Mar 26;9(11):3647–55. doi: 10.1002/cam4.3023 (PMC7286455; doi:10.1002/cam4.3023)
Supplement: Supplementary file 5 — Supplementary Material [file CAM4-9-3647-s005.docx]

Figure S1a and S1b. Overall survival (OS) and progression-free survival (PFS) analysis for the AML-M5 children in two age groups. In comparison to the children>3 years group, significant poorer OS (*P*=0.009) and PFS (*P*=0.004) were found in the infants (age≤3 years) group.

Figure S2a and S2b. Overall survival (OS) and progression-free survival (PFS) analysis for the AML-M5 children with hyperleukocytosis or not. In comparison to the children without hyperleukocytosis, significant poorer OS (*P*<0.001) and PFS (*P*<0.001) were found in the patients suffered from hyperleukocytosis.
